# Tribological Properties of Spark Plasma Sintered Al-SiC Composites

**DOI:** 10.3390/ma13214969

**Published:** 2020-11-04

**Authors:** Beata Leszczyńska-Madej, Marcin Madej, Dariusz Garbiec

**Affiliations:** 1 Faculty of Non-Ferrous Metals, AGH University of Science and Technology, Mickiewicza 30 Ave., 30-059 Krakow, Poland; 2 Faculty of Metals Engineering and Industrial Computer Science, AGH University of Science and Technology, Mickiewicza 30 Ave., 30-059 Krakow, Poland; mmadej@agh.edu.pl; 3 Łukasiewicz Research Network – Metal Forming Institute, 14 Jana Pawla II St., 61-139 Poznan, Poland; dariusz.garbiec@inop.lukasiewicz.gov.pl

**Keywords:** tribological properties, Al-SiC composites, spark plasma sintering, wear mechanisms, coefficient of friction, weight loss

## Abstract

The paper presents the results of research on the tribological properties of spark-plasma-sintered Al-SiC composites. Composites with contents of 50 and 70 wt.% SiC were prepared. The sintering process was carried out using an HP D 25/3 spark plasma sintering furnace under vacuum, at the sintering temperature of 600 °C and compaction pressures of 50 and 80 MPa, respectively. The heating rate was 100 °C/min and the holding time was 10 min. Composites with a density of 91–100% were obtained. The tribological properties of the composites were evaluated based on weight loss and the coefficient of friction using a block-on-ring tribotester. Along with the weight percentage of SiC and compaction pressure, the sliding distance, and load during the tribological test were considered. Both the weight percentage of SiC and compaction pressure affected the tribological behavior of Al-SiC composites. It was found that the wear resistance was higher when a lower compaction pressure and a smaller amount of reinforcing phase (50 wt.%) were used.

## 1. Introduction

Aluminum (Al) powders are commonly used to produce composites reinforced with hard ceramic particles. The basic problem that occurs when sintering Al powder is its strong affinity for oxygen, resulting in the surface of the powder particles being covered with a thin film of oxide that creates a barrier to forming a permanent intermetallic bond between the sintered powder particles. In addition, oxygen reacts with some ceramics to form intermetallic phases affecting the quality of the metal–ceramic interface and, thus, the final properties of the composite [1,2,3]. Al reacts with oxygen at room temperature, which results in the formation of a permanent aluminum oxide (Al_2_O_3_). The oxide film is continuous, pore-free, and prevents further oxidation of Al. For this reason, a continuous film of Al_2_O_3_ can be treated as a positive factor; however, during sintering, this thermodynamically stable oxide film covering individual Al particles acts as a barrier to the formation of metal-to-metal bonds, necessary for the formation of necks between the particles, and their growth, as well as to the volumetric diffusion processes responsible for shrinkage during sintering [2]. The sintering point of Al_2_O_3_ powder is much higher than those of Al-based powders [3]. Therefore, to ensure an effective sintering process, the oxide film must be destroyed. The liquid phase is beneficial to the sintering of Al as it reduces the integrity of the oxide film formed on the surface of Al particles by penetrating the interphase boundary between the oxide film and the metal [2].

The sintering process parameters have a strong influence on the change in the dimensions of the sintered compacts during the process and the creation of additional process products resulting from the reaction between the components of the sintered material, as well as the gas coming from the atmosphere. Thus, this influences changes in the microstructure of the sintered material. In the classical powder metallurgy route, nitrogen is the most favorable atmosphere for sintering Al [3,4,5,6]. Al-based and Al alloy-based composites reinforced with ceramic particles are characterized by low density, high specific strength, good corrosion resistance, as well as high hardness and good abrasion resistance [7,8,9]. These properties are largely determined by the content of ceramic particles, as well as by their distribution and size [10,11,12,13,14].

Many researchers have studied the effect of SiC reinforcement on the wear behavior of Al-SiC composites. An increase in wear resistance was observed with an increase in SiC content [15,16,17,18,19,20,21]. Chen et al. [16] investigated the impact of the SiC reinforcement volume fraction on the wear properties. They concluded that the wear rates decreased with volume fraction, whereas the coefficient of friction increased. By the same token, Gosh et al. [17] reported a decrease in the wear rates as the SiC volume fraction was increased in LM6 Al-based composites produced by means of stir casting. Smrutiranjan et al. [18] presented the results of the wear behavior of Al-SiC composites. The conclusion they reached was that the wear increases with the applied load, and the wear mechanism might also change. The authors of work [19] observed that adding copper to the Al matrix may enhance the tribological properties. Leszczyńska [20] presented the results of research on the tribological properties of Al-SiC composites sintered in a nitrogen atmosphere at temperatures of 580 °C and 620 °C. The obtained results indicated that the weight loss and the coefficient of friction were lower after sintering at 620 °C than after sintering at 580 °C. Martin et al. [21] investigated the influence of the temperature on the wear resistance of a AA2618 reinforced with 15 vol% SiC. They proved that the wear resistance in the mild wear region improved by a factor of two, while the transition temperature was approximately 50 °C higher in the composite material due to the SiC particle addition.

Al-based composites reinforced with ceramic particles are produced by many methods, both by casting and by classical powder metallurgy techniques [1,2,3,4,6,7,8,10,22,23,24]. One of the advanced techniques of powder metallurgy used to produce composites is spark plasma sintering (SPS). This technique belongs to the group of field assisted sintering techniques (FAST), and its characteristic feature is the use of pulse DC to directly heat the compacted powder and to obtain plastic deformation. The spark discharges in the gaps, generated in the initial sintering stage, cause a local increase in temperature on the surface of the powder particles, which leads to the evaporation of most oxides, thus revealing clean metallic surfaces that are the pathway for diffusion. As a result, diffusion phenomena are activated at a lower temperature and their intensity is much higher [25,26,27].

In the literature on composites based on Al or Al alloys, there is little information on the SPS process, especially concerning composites with a high content of the SiC reinforcing phase. Literature data neither provide uniform data for the sintering of such composites by using the SPS technique nor the tribological properties. Leszczyńska et al. [28] presented the results of a study of the microstructure and selected properties of SPSed Al matrix composites reinforced with different fractions of SiC particles and sintered at 580 °C and 600 °C. The obtained results show that the higher sintering temperature had a positive effect both on the microstructure and mechanical properties. The authors found nanometric-size Al_2_O_3_ in the microstructure and also the presence of Al_4_C_3_ carbide. The formation of Al_4_C_3_ carbide in sintered Al-SiC composites was also revealed by Zhang et al. [29]. Van Trinh et al. [30] studied the effect of the oxidation of SiC particles on the wear behavior and mechanical properties of SiC-AA6061 composites fabricated using SPS. The results revealed an improvement in the interfacial bond strength between AA6061 and the oxidized Al-SiC due to the formation of an MgAl_2_O_4_ continuous phase. Additionally, the coefficient of friction and specific wear rate were reduced, which is explained by the increase in the mechanical properties and also by the transition of the wear mechanism from a combination of adhesive and fatigue wear to abrasive wear. In [31], the authors presented results concerning the influence of the sintering temperature of 50 vol.% Al-SiC composites on the thermal properties and bending strength of such composites. The results reveal that the composites fabricated by sintering at the temperature of 520 °C for 8 min under 50 MPa have the best properties, which is explained by the nearly full density of such composites.

The results presented herein provide information about the tribological properties and wear mechanisms depending on the amount of reinforcing phase (SiC) and SPS process parameters. Due to different amounts of the SiC phase (50 and 70 wt.%) and different compaction pressures (50 and 80 MPa), the investigated composites have different microstructures and properties, which has a direct impact on the tribological properties of these materials. 

## 2. Materials and Methods 

**Figure 1:**
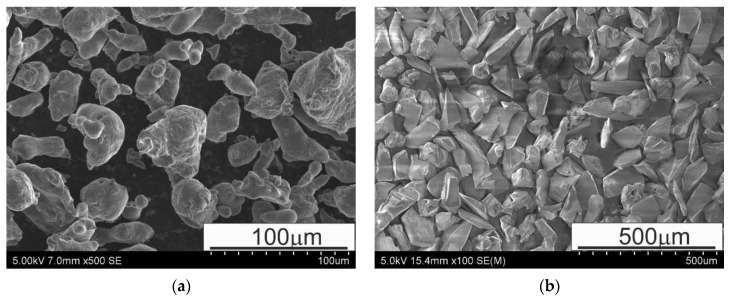
SEM micrographs of powders: (**a**) Al (<63 µm) and (**b**) SiC (60–80 µm); SEM.

The fabricated composite samples were based on Al with different amounts of reinforcement in the form of SiC particles (50 and 70 wt.%). The process of air gas atomization was employed to produce the Al powder particles (Benda Lutz, Skawina, Poland), which had an average size of <63 µm. Nearly 70% of powder particles in the powder fraction had sizes within the 32–63 μm range, whereas the remaining ones were below 32 μm. SiC powder particles (Karborund Sp. z o.o, Gliwice, Poland) with an average size of 60–80 µm in the form of particulates were added in quantities of 50 and 70 wt.% to the alloy matrix. The morphology of the starting powder materials can be seen in Figure 1.

A homogenous powder mixture was produced by mixing pure Al powder with the SiC powder in a Turbula T2F shaker-mixer for 30 min. The sintering process was carried out using an HP D 25/3 (FCT Systeme, Frankenblick, Germany) spark plasma sintering furnace under a vacuum of 5 × 10^−2^ mbar at the sintering temperature of 600 °C, and compaction pressures of 50 and 80 MPa. The temperature was monitored by an optical pyrometer through a nonthrough hole in the upper punch. The distance between the point of temperature measurement and the sample was 5 mm. The heating rate was 100 °C/min and the holding time was 10 min. The on:off ratio of pulsed current was set at 125:5 (ms/ms). Cylindrical-sintered compacts with dimensions of 40 mm in diameter and 10 mm in thickness were produced.

The microstructure of the composites was investigated with a GX 51 (Olympus, Tokyo, Japan) light microscope (LM) and an SU-70 (Hitachi, Tokyo, Japan) Schottky-type electron gun scanning electron microscope (SEM) with a Thermo Scientific NORAN System 7 X-ray microanalysis system (EDS) (Thermo Fisher Scientific, Waltham, MA, USA). Examinations were carried out at various magnifications. The Brinell hardness (HB) was measured using a 2.5 mm-diameter carbide ball and a force of 612.92 N applied on mirror-shine-polished specimen surfaces, and the Brinell hardness (HB) was measured. 

**Figure 2:**
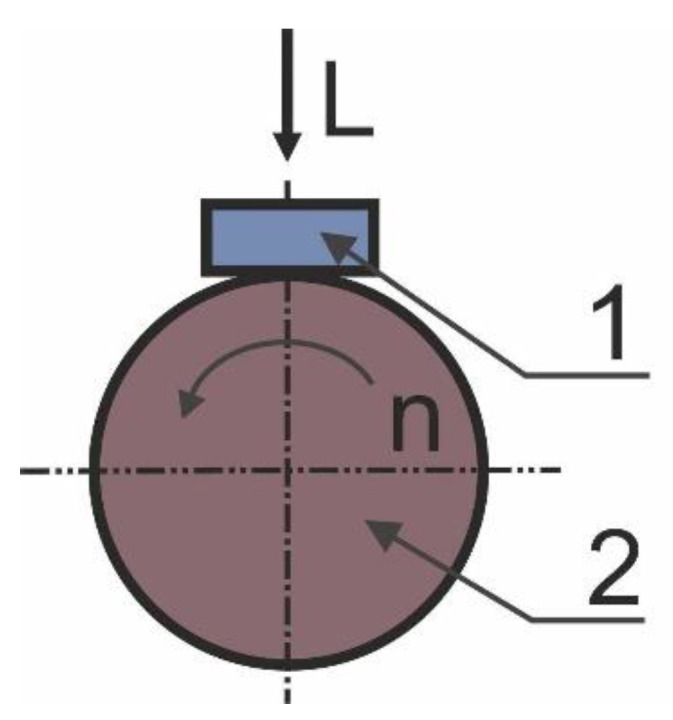
Schematic view of block-on-ring tester: 1—specimen, 2—rotating ring.

The tribological properties of the Al-SiC composites were determined by testing 20 × 4 × 4 mm specimens in a block-on-ring T-05 (ITEE, Radom, Poland) tribotester (Figure 2) under dry friction conditions and at ambient temperature. Thanks to this tester, tests can be performed according to the methods set out in the ASTM D 2714, D 3704, D 2981, and G 77 standards. For each variant of the studied material, a minimum of three tribological tests were carried out.

The specimen (1) was secured in a specimen holder equipped with a hemispherical insert that ensures appropriate contact between the specimen and a rotating ring (2). The specimen’s wear surface was situated perpendicular in relation to the pressing direction. The double lever system transmits load L by pressing the specimen against the ring to the accuracy of ±1%. The ring is rotated at a perpetual rotating speed. A counter-specimen (a rotating ring) 49.5 mm in diameter made of heat-treated 100Cr6 bearing steel having a hardness of 53 HRC was employed. All the dry-condition wear tests were performed at a perpetual rotating speed amounting to 136 rpm throughout the test. The load throughout the test was also maintained at the same level; two loads were applied: 100 and 200 N. Before the test, samples were prepared in terms of surface quality (an appropriate roughness—below 1 µm) and thoroughly degreased by rinsing in a Struers degreasing agent; after drying, they were weighed and tested immediately afterward. After the test, the sample was also washed, dried, and weighed. The weight loss was determined from the difference in weight. As the initial samples are characterized by different weights, in order to accurately characterize the differences, the weight losses are converted to %. Changes in the coefficient of friction over time were ascertained for the run-in period (25 m) and the overall sliding distance (1000 m). The friction force and load were measured to calculate the coefficient of friction. The wear surface was analyzed by a scanning electron microscope in order to ascertain the wear mechanisms.

## 3. Results and Discussion

### Microstructure and Properties Examinations

**Figure 3:**
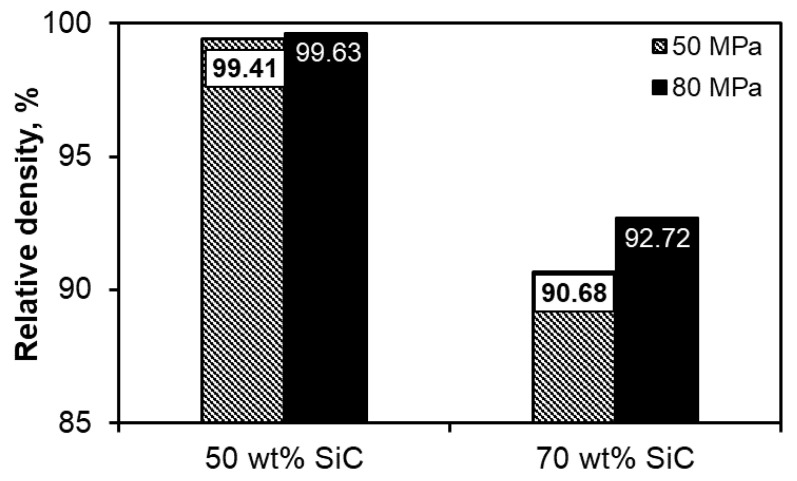
Relative densities ascertained by Archimedes method.

Figure 3 illustrates the influence of the used weight content of the SiC phase on the relative density ascertained by means of the Archimedes method. The determined theoretical density of the composites was as follows: ρ = 2.95 and 3.06 g/cm^3^ for Al-50SiC and Al-70SiC, respectively. In the case of composites containing 50 wt.% SiC, regardless of the compaction pressure used, a material with a density close to the theoretical one was obtained. In the case of composites containing 70 wt.% SiC, the sintered compacts produced at the higher compaction pressure (80 MPa) were characterized by a higher density. The determined densities were 90.68% and 92.72%, respectively, for the sintered compacts produced using the compaction pressures of 50 and 80 MPa.

**Figure 4:**
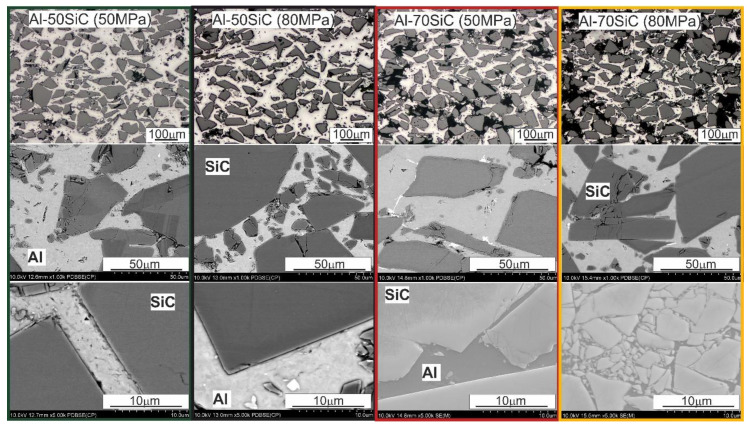
Microstructure of spark plasma sintered Al-SiC composites.

It was possible to distribute the reinforcing phase particles evenly and prevent SiC agglomerates from forming due to the use of a SiC reinforcing phase having a particle size nearly identical to the matrix material, as well as owing to mixing the powders additionally in a Turbula T2F shaker-mixer. Locally, especially in the composites with 70 wt.% SiC, pores/voids are visible. They can also be SiC particle chipping, formed during preparation of the metallographic specimens. Regardless of the compaction pressure and SiC content, the sintered compacts were characterized by a good connection at the Al/SiC interface, which is particularly well-visible in the micrographs taken at high magnification (Figure 4).

**Figure 5:**
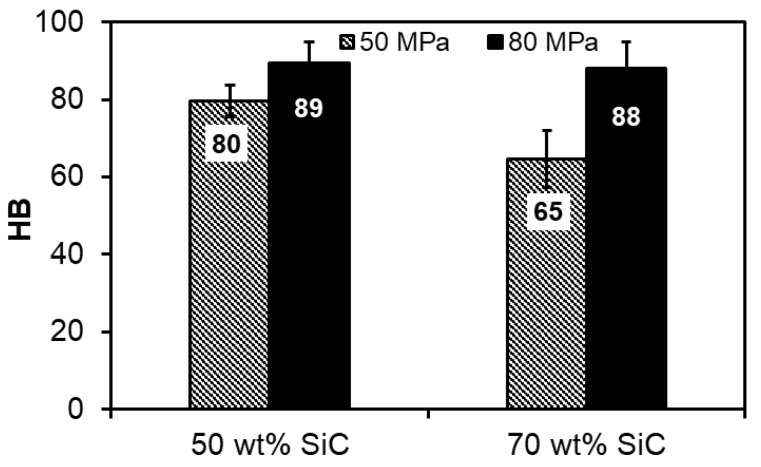
Hardness of spark plasma sintered Al-SiC composites.

Figure 5 presents the evolution of the Brinell hardness in the Al-SiC composites, respectively, as a function of the SiC wt.% and the applied compaction pressure. The obtained results correlate with the density measurements. It can be seen that using a higher compaction pressure resulted in a higher hardness. Additionally, the hardness of the composites with the addition of 50 wt.% SiC was higher than the hardness of the sintered compacts with 70 wt.% SiC. In the case of the sintered compacts with 50 wt.% SiC, the higher compaction pressure resulted in an 11% increase in hardness, and 35% in the case of composites with 70 wt.% SiC. The hardness improved by applying a higher compaction pressure. It was achieved due to the higher concentration of reinforcing phase particles in the matrix, which reduces the effective distance between the SiC particles. This results in better strength properties as the particles form a sustained skeleton of the strengthening phase.

**Figure 6:**
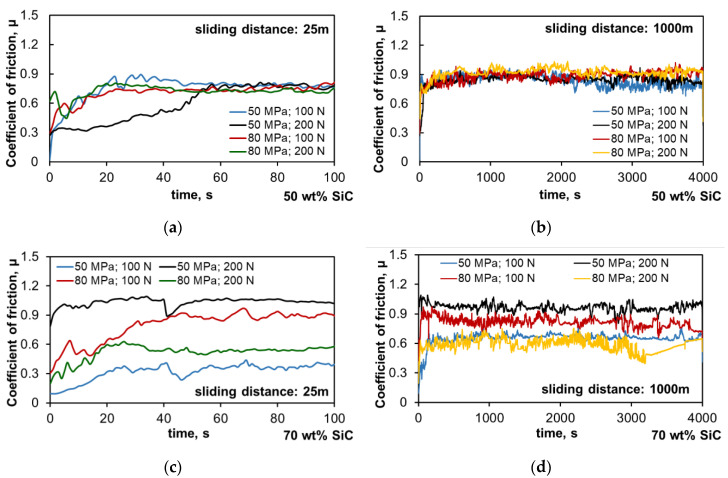
Coefficient of friction as a function of time: (**a**) Al-50SiC, run-in period—sliding distance 25 m, (**b**) Al-50SiC, whole test—sliding distance 1000 m, (**c**) Al-70SiC, run-in period—sliding distance 25 m, (**d**) Al-70SiC, whole test—sliding distance 1000 m.

**Figure 7:**
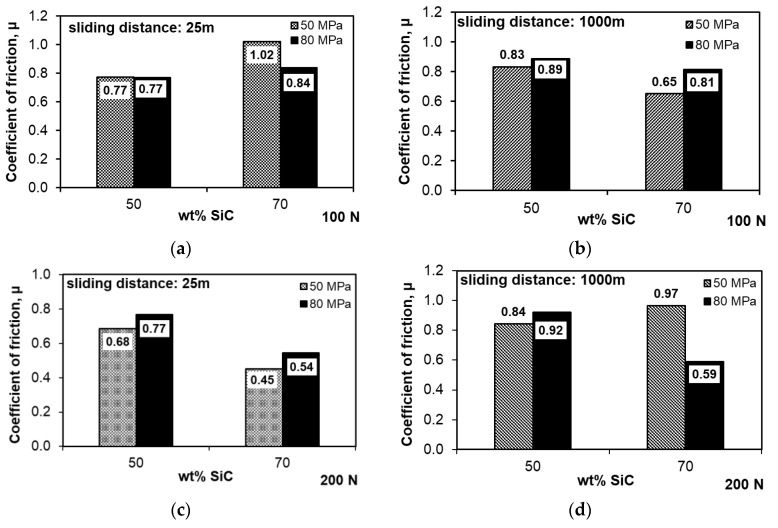
Variation in coefficient of friction in composites: (**a**,**b**) Load 100 N; (**c**,**d**) load 200 N.

**Figure 8:**
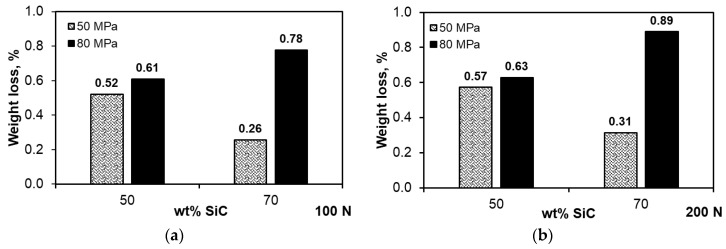
Variation in weight loss: (**a**) Load 100 N, (**b**) load 200 N.

**Figure 9:**
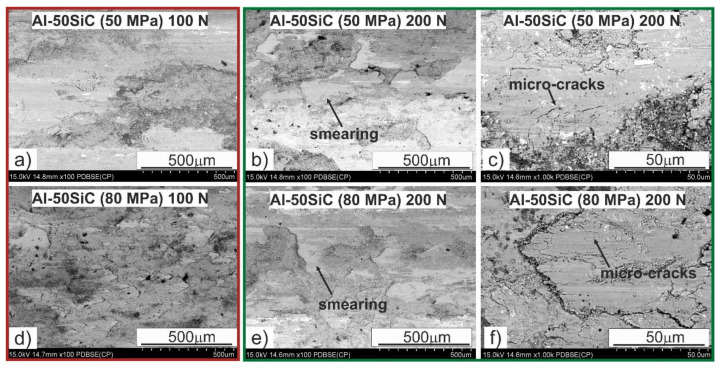
Al-50SiC specimen surfaces after tribological tests.

**Figure 10:**
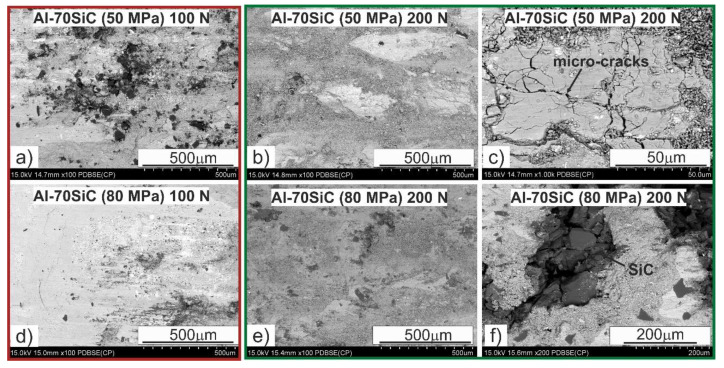
Al-70SiC specimen surfaces after tribological tests.

The tribological properties are presented in Figure 6, Figure 7, Figure 8, Figure 9 and Figure 10. In Figure 6 and Figure 7 are graphs presenting changes in the coefficient of friction as a function of time for the chosen materials under dry friction conditions. The changes in the coefficient of friction in the initial stage for the sliding distance of 25 m and the entire sliding distance of 1000 m are presented.

The changes in the coefficient of friction presented in Figure 6a,c during the tribological test in the run-in period (100 s), which correspond to the run-in of the surface after start-up, prove the significant differences in the initial stage of wear, especially in the case of the composites with the high amount of SiC. The Al-SiC composites fabricated with the lower compaction pressure of 50 MPa showed significant differences in the initial course of friction depending on the applied load, while those sintered under the compaction pressure of 80 MPa had an almost identical linear course, and stabilization of the coefficient of friction occurred much earlier than in the case of the other tested specimens. This proves the better fixation of the reinforcing phase in the matrix resulting from the application of higher compaction pressure, regardless of the increased load during the test. By increasing the amount of strengthening phase in the composite to 70 wt.%, significant changes in the coefficient of friction in the run-in period are observed, and it can be concluded that they are characteristic of individual shapes depending on the production parameters. The course of changes for the composites sintered under the compaction pressure of 50 MPa depends on the applied load, which indicates the possibility of SiC chipping already in the initial stage, while the sintered compacts obtained under the compaction pressure of 80 MPa show an opposite dependence. Observing the changes in the coefficient of friction curves during the entire test, a similar relationship was noticed—the increased load had a positive effect on the friction course. The key parameter may be the very high amount of strengthening phase in the form of SiC particles, which can be blocked by the easier smearing of the matrix on the surface. However, when interpreting the results for composites with such a high amount of the hard phase, its distribution in the specimen volume and the size of its clusters should be taken into account. It is visible that the 50 wt.% amount of the strengthening phase had a positive effect on the frictional course, and the curves had a similar course with a similar coefficient of friction. After analysis of the course of changes in the coefficient of friction presented in Figure 6b, it can be seen that after 3000 s, the characteristics of the curves changed; the composites sintered at the compaction pressure of 50 MPa were characterized by a stable course of changes, while for the composites sintered at the compaction pressure of 80 MPa, the coefficient of friction increased slightly, which may indicate a negative impact of the higher compaction pressure on the fixation of the reinforcing phase in the matrix and the generation of stresses resulting from its interaction. Observations of the character of changes in the coefficient of friction, both in the initial stage and in the entire test, translate into the determined mean coefficient of friction (Figure 7). Considering these results, it can be seen that the coefficient of friction of the sintered compacts with the 50 wt.% SiC content was usually higher than that for the sintered compacts with the 70 wt.% SiC content, which results from the role of the Al matrix in the friction process. The presence of Al in the friction pair changed the mechanisms occurring there; the role of adhesion increased, which strongly influenced the increase in the coefficient of friction. Exceptions were the specimens with the 70 wt.% SiC content, sintered at the 80 MPa compaction pressure, which were characterized by a lower density. The coefficient of friction determined during the run-in period under the load of 100 N and the entire test under the load of 200 N was higher. Apart from these two cases, increasing the compaction pressure applied during sintering adversely affected the coefficient of friction.

Figure 8 shows the weight losses recorded during the dry tribological test at a distance of 1000 m under variable loads of 100 and 200 N. The aspects determining the obtained results were both the parameters of their production and the load in the friction pair. The composites with the 50 wt.% SiC content were slightly sensitive to the increase in the load from 100 to 200 N. Their wear resistance decreased slightly by 0.05% (Figure 8). A factor that largely determines the wear resistance is the compaction pressure used during sintering. More beneficial than wear resistance was the lower compaction pressure of 50 MPa; this was due to the lower stresses, especially in the SiC particles, which can cause them to crack and chip during the test under higher loads. Increasing the content of the strengthening phase had a positive effect on the wear resistance (Figure 8) only in the case of the composites sintered at the compaction pressure of 50 MPa; increasing this pressure to 80 MPa caused a significant increase in weight loss, and thus resistance to abrasive wear. The lower densification pressure turned out to be more favorable for the tribological properties, regardless of the applied load in the pair. In the case of the composites with the high content of strengthening phase, its distribution in the matrix, constituting only 30%, as well as the distribution of pores, became important. The microstructures of the composites with the 70 wt.% SiC content shown in Figure 4 indicate that there was a significant proportion of boundaries between SiC particles, and the manufacturing conditions used might have been insufficient to obtain a permanent bond, while the applied higher compaction pressure might have caused stresses in these areas of the microstructure. This translated into significant weight loss with a relatively low coefficient of friction (Figure 7b,d). Pores were also concentrated in the most common areas of the SiC particles, which facilitated cracking, especially in the test under the load of 200 N.

Analysis of the surface after friction presented in Figure 10 allows one to determine the wear mechanisms occurring in the friction pair on the surface of the tested composite in contact with a steel counter-sample with a hardness of about 53 HRC. The wear mechanisms mainly depend on the content of the strengthening phase, the compaction pressure used during sintering, and to the least extent on the applied load in the friction pair. In the composites with the 50 wt.% SiC content, we can mainly observe smearing of the soft Al matrix on the friction surface, isolating the SiC particles from contact with the counter-sample. This phenomenon occurred during the run-in period (Figure 6a,c, and Figure 7a,c) and led to subsequent stabilization of the friction trajectory, especially under the load of 100 MPa. Increasing the load caused the appearance of microcracks, transverse to the direction of friction. Increasing the compaction pressure during sintering changed the route of matrix smearing; it did not run as freely as in the case of the composites sintered at the compaction pressure of 50 MPa. Clear delamination was formed due to the differences in the height of individual areas (Figure 9f), which caused intensification of the adhesion process and a characteristic adhesive bond during the tribological contact between steel and Al. Al_2_O_3_ were also observed on the surface of the Al matrix, which confirms the presence of a wear mechanism involving oxidation, typical for this type of material, regardless of the friction conditions. In the case of the composite sintered under the compaction pressure of 80 MPa, oxides concentrated on the edge of the smearing Al matrix (Figure 9f). Only a small amount of abrasive wear can be seen on the worn surfaces, mainly small scratches within the Al, which proves that the dominant mechanism was plastic deformation, which removed the traces of abrasive wear (scratching and grooving) resulting from the presence of chromium carbides on the counter-sample surface.

Increasing the content of the strengthening phase to 70 wt.% changed the wear mechanisms during the tribological test (Figure 10). The smearing of Al was still observed, but on a smaller scale due to its lower content in the material. When we look at the surface morphologies of the composites sintered at the compaction pressure of 50 MPa, smearing Al on it was easier; however, there were clear boundaries between the Al regions related to the presence of local SiC clusters (Figure 4) and the formation of an oxide layer at the boundaries between these regions.

The microcracks are also characteristic, both in the direction of friction and transverse; they were the result of adhesive wear. There are visible effects of abrasive wear, also related to the smaller amount of Al on the surface of the composite during tribological contact, which were not so effectively removed by the plastic deformation of Al. Attention should also be paid to the large gaps present on the surface after friction of the Al-70SiC composite sintered under the compaction pressure of 80 MPa. Chipping was located in the SiC areas, which confirms the previous discussions regarding maintaining the coefficient of friction in these materials. These gaps were related to the presence of boundaries between the SiC particles that had not been sintered, and the increased compaction pressure additionally caused stresses in these areas.

## 4. Conclusions

The following conclusions can be made from the conducted study:

1. The effective distance between the SiC particles was reduced owing to the higher concentration of strengthening-phase particles in the matrix, resulting from a higher applied compaction pressure, which improved not only the density but also the hardness.
2. During the run-in period, significant differences in the coefficient of friction were found, especially in the case of the composites with 70 wt.% SiC. In the composites sintered with the lower compaction pressure of 50 MPa, the initial course of friction depended on the applied load, while in those sintered under 80 MPa, an almost identical linear course and stabilization of the coefficient of friction occurred, which prove the better fixation of the reinforcing phase in the matrix, regardless of the increased load during the test.
3. The determined coefficient of friction for the sintered compacts with the 50 wt.% SiC content was usually higher than that for the sintered compacts with the 70 wt.% SiC content, which resulted from the role of the Al matrix in the friction process. The presence of Al in the friction pair changed the mechanisms occurring there; the role of adhesion increased, which influenced the increase in the coefficient of friction. Additionally, increasing the compaction pressure applied during sintering adversely affected the coefficient of friction.
4. The wear mechanisms of the investigated composites mainly depended on the content of the strengthening phase, the compaction pressure used during SPS, and to the least extent on the applied load in the friction pair.
